# Effect of Encapsulated Purple Garlic Oil on Microvascular Function and the Components of Metabolic Syndrome: A Randomized Placebo-Controlled Study—The ENDOTALLIUM Study

**DOI:** 10.3390/nu16111755

**Published:** 2024-06-03

**Authors:** Nuria Bara-Ledesma, Judith Jimenez-Esteban, Martin Fabregate, Rosa Fabregate-Fuente, Leandro Javier Cymberknop, Purificacion Castillo-Martinez, Maria Teresa Navarro-Fayos, Vicente Gomez del Olmo, Jose Saban-Ruiz

**Affiliations:** 1 Internal Medicine Department, Hospital Universitario Ramón y Cajal, IRYCIS, 28034 Madrid, Spain; 2 Faculty of Medicine and Health Sciences, Universidad de Alcalá (UAH), 28805 Alcalá de Henares, Spain; 3 Group of Research and Development in Bioengineering (GIBIO), Universidad Tecnológica Nacional, Buenos Aires C1179AAQ, Argentina; 4 R&D Department, COOPAMAN S.C.L., Las Pedroñeras, 02006 Cuenca, Spain; 5 Food Industries Department, AINIA, 46980 Paterna, Spain

**Keywords:** garlic, purple garlic oil, microvascular function, metabolic syndrome, inflammation, organosulfur compounds

## Abstract

Endothelial dysfunction (ED) is associated with progressive changes contributing to clinical complications related to macro- and microvascular diseases. Garlic (*Allium sativum* L.) and its organosulfur components have been related to beneficial cardiovascular effects and could improve endothelial function. The ENDOTALLIUM Study aimed to evaluate the effect of the regular consumption of encapsulated purple garlic oil on microvascular function, endothelial-related biomarkers, and the components of metabolic syndrome (MetS) in untreated subjects with cardiometabolic alterations. Fifty-two individuals with at least one MetS component were randomized (1:1) in a single-center, single-blind, placebo-controlled, parallel-group study. The participants received encapsulated purple garlic oil (n = 27) or placebo (n = 25) for five weeks. Skin microvascular peak flow during post-occlusive reactive hyperemia significantly increased in the purple garlic oil group compared to the placebo group (between-group difference [95%CI]: 15.4 [1.5 to 29.4] PU; *p* = 0.031). Likewise, hs-CRP levels decreased in the purple garlic group compared to the control group (−1.3 [−2.5 to −0.0] mg/L; *p* = 0.049). Furthermore, we observed a significant reduction in the mean number of MetS components in the purple garlic group after five weeks (1.7 ± 0.9 vs. 1.3 ± 1.1, *p* = 0.021). In summary, regular consumption of encapsulated purple garlic oil significantly improved microvascular function, subclinical inflammatory status, and the overall MetS profile in a population with cardiometabolic alterations.

## 1. Introduction

The endothelium is a metabolically active organ that plays a pivotal role in the regulation of vascular tone [1]. Endothelial function refers to the ability of the endothelium to synthesize and release factors that can induce a direct relaxation of smooth muscle cells within the vascular wall [2]. Endothelial dysfunction (ED) is associated with progressive changes including pro-inflammatory, pro-oxidant, proliferative, and prothrombotic status, as well as an abnormal modulation of vascular tone [3]. ED not only preludes the onset of atheromatous plaque, but also contributes to lesion development and later clinical complications related to macro- and microvascular diseases [1].

Several approaches have been proposed to measure the patho-physiological function of the vascular endothelium, including circulating biomarkers and non-invasive methods [4]. Most studies aimed at studying endothelial dysfunction have focused on large arteries, mainly assessing the flow-mediated dilatation of the brachial artery. Although microvascular dysfunction has been less studied, it is known that it precedes the morphological alterations and endothelial damage in large arteries [1]. The assessment of skin post-occlusive reactive hyperemia (PORH) coupled with laser Doppler flowmetry (LDF) can be used as a sensitive indicator of microvascular dysfunction [5]. Impairment in microvascular perfusion has been associated with pathological conditions such as cardiovascular disease, erectile dysfunction, chronic kidney disease, diabetes mellitus, obesity, or hypertension [6,7,8,9]. The three latter conditions are components of metabolic syndrome (MetS), a cluster of cardiometabolic abnormalities that is strongly associated with an increased risk for developing type 2 diabetes mellitus and cardiovascular disease [10]. Previous studies have shown that pharmacological and non-pharmacological therapeutic interventions to reduce the risk of cardiometabolic diseases have a beneficial impact on the microvasculature [11].

The Mediterranean diet, which typically includes a high consumption of olive oil, nuts, and vegetables such as garlic or onion [12], has been consistently associated with positive effects on cardiovascular health [13] and endothelial function [14]. In particular, garlic (*Allium sativum* L.) has been shown to have lipid-lowering, hypoglycemic, antihypertensive, and antithrombotic effects [15]. A number of studies, both in vitro and in animal models, have associated garlic’s beneficial cardiovascular effects with sulfur compounds [16,17]. Specifically, garlic oil contains organosulfur compounds such as diallyl sulfide (DAS), diallyl disulfide (DADS) and diallyl trisulfide (DATS) [18], which form from unstable allicin and other thiosulfonates [19]. In this regard, it has been reported that the purple garlic ecotype contains a higher concentration of organosulfur compounds than the white garlic ecotype [20]. Nevertheless, the digestive tract could compromise the bioavailability of these compounds, degrading them before they can be absorbed in the intestine. In this regard, encapsulation has emerged as a proven strategy for enhancing the absorption of edible oils by the intestinal epithelium [21]. However, few clinical studies have evaluated the effects of garlic oil or its bioactive compounds on the microvascular function in adults at risk of cardiovascular disease [22].

In this context, the ENDOTALLIUM Study aims to evaluate the effect of the regular consumption of encapsulated purple garlic oil on microvascular function, endothelial-related biomarkers, and the components of MetS in untreated subjects with cardiometabolic alterations.

## 2. Materials and Methods

### 2.1. Design

The ENDOTALLIUM Study was a 5-week, prospective, randomized, single-center, single-blind, placebo-controlled, parallel-group study, which was conducted at the Endothelial and Cardiometabolic Medicine Unit (Internal Medicine Department) of Hospital Universitario Ramón y Cajal, Madrid, Spain.

### 2.2. Study Population

The study was advertised for enrollment through posters placed in public areas of Hospital Universitario Ramón y Cajal between September 2017 and January 2019. Eligible subjects were men and women aged between 18 and 65 years, who met at least one of the following metabolic syndrome components (NCEP/ATP III criteria) [23]: (i) abdominal obesity, defined as a waist circumference ≥ 102 cm for males and ≥88 cm for females; (ii) triglycerides ≥ 150 mg/dL; (iii) HDL-cholesterol < 40 mg/dL in men, and <50 mg/dL in women; (iv) systolic blood pressure ≥ 130 mmHg, or diastolic blood pressure ≥ 85 mmHg; (v) fasting plasma glucose ≥ 100 mg/dL. We excluded subjects with history of cardiovascular disease, or those who were undergoing pharmacological treatment for diabetes, hypertension, or dyslipidemia. Subjects with body mass index (BMI) ≥ 40 kg/m^2^ or <18.5 kg/m^2^ were also excluded. Any other conditions that could interfere with study development, such as pregnancy, alcohol or drug abuse, heavy smoking (>20 cigarettes/day), known allergies to garlic or to the excipient, mental disorders, anemia, gastrointestinal, kidney, pulmonary, or liver diseases were excluded. Additionally, subjects who reported daily intake of antioxidant or garlic-derived nutritional supplements, as well as high dietary consumption of garlic (≥2 garlic cloves/day), vegetables (≥5 servings/day), or nuts (≥14 servings/week) were excluded. Subjects planning to significantly modify their lifestyle habits (physical activity, diet, or smoking habits) during the course of the study were considered ineligible.

### 2.3. Investigational Product

The investigational product was encapsulated purple garlic oil. Purple garlic bulbs were harvested by COOPAMAN S.C.L. in the area of Las Pedroñeras (Spain), the only European region with a protected geographical indication for garlic. The purple garlic variety from Las Pedroñeras is an autochthonous ecotype with high content of organosulfur compounds, especially allicin [24]. The oil was extracted by steam distillation (GLOBALAB I+D S.L.) with a yield of 0.20% (0.20 g oil/100 g garlic). The purple garlic oil had the following concentrations for the organosulfur compounds of interest: 96.97 mg/g of DADS, 6.78 mg/g of DATS, and 9.50 mg/g of DAS. Quantification was carried out by high-performance liquid chromatography (HPLC) at Centre D’investigacio I Control Alimentari S.L. (Barcelona, Spain), using H_2_O extraction with dilute HCl (0.05 N), purification with Setpack (columns), and a C18 chromatographic column. The mobile phase was a mixture of water and acetonitrile, eluted at a flowrate of 1 mL/min. The UV fluorescence detector was set at 273 nm.

The purple garlic oil underwent two encapsulation processes (AINIA Centro Tecnológico, Paterna, Spain): first, it was microencapsulated in a ratio of 4 mg of oil to 16 mg of corn starch, and then it was macroencapsulated with a gelatin coating and filled with corn starch as an excipient. Each capsule had a total weight of approximately 350 mg, of which 4 mg was purple garlic oil. As a placebo, we used capsules with a similar appearance that contained only corn starch.

### 2.4. Study Intervention and Procedures

The study protocol and the informed consent form were approved by the institutional Research Ethics Committee (reference: 174/17). Before any trial-related activities, all participants were informed about the study protocol and gave their written informed consent. Medical history, lifestyle questionnaires, and laboratory parameters were used to assess their eligibility. For those who met the selection criteria, the randomization took place on the same day as the screening visit. Participants were randomly assigned (1:1) to receive one of the following two interventions: encapsulated purple garlic oil or placebo capsules for five weeks. Participants remained blinded to the allocation and were instructed to maintain their habitual diet and physical activity throughout the study. During the 5-week intervention period, two follow-up telephone calls (at weeks 1 and 2) were made to collect reports of any adverse events and to confirm correct intake of the investigational product.

The target dose for the purple garlic oil arm was 8 mg daily, taking into account the recommended daily amount of equivalent raw garlic consumption (1–2 cloves of garlic) [25]. The target dose was reached following a dose escalation schedule. The starting dose was 4 mg/day (one capsule) during the first week, and then the dose was increased to 8 mg/day (one capsule twice daily) for the remaining four weeks. Each intake was accompanied by a main meal. A total treatment period of five weeks was chosen, as it is in line with previous human intervention studies on garlic [26].

### 2.5. Study Variables

#### 2.5.1. Clinical Variables

Height, body weight and waist circumference were measured, and BMI (kg/m^2^) was calculated. Systolic (SBP) and diastolic (DBP) blood pressure were measured in a sitting position after five minutes of rest, using an automated sphygmomanometer (Omron 705CP, Kyoto, Japan). Relevant dietary habits (e.g., garlic, fruits, and vegetables consumption) and smoking status were recorded at baseline. The number of Met-S parameters was collected for each participant, ranging from one to five. The physical activity rating scale (PA-R, from 0 to 7 points) [27] was used to classify the baseline physical activity status as follows: low (PA-R from 0 to 1 points); moderate (PA-R from 2 to 3 points); or high (PA-R ≥ 4 points).

#### 2.5.2. Laboratory Parameters

Overnight fasting blood samples and first-void urine samples were collected and processed. Concentrations of fasting plasma glucose (mg/dL), and lipid parameters, including total cholesterol (mg/dL), LDL-cholesterol (mg/dL), HDL-cholesterol (mg/dL), and triglycerides (mg/dL), were analyzed in the certified local laboratory using standard procedures.

#### 2.5.3. Circulating Biomarkers

Highly sensitive C reactive protein (hs-CRP) (mg/L) was assessed using nephelometric method (Siemens, Munich, Germany). Serum vascular cell adhesion molecule-1 (VCAM-1) (ng/mL), plasminogen activator inhibitor-1 (PAI-1) (ng/mL), and urinary F2-isoprostanes were measured using commercial enzyme-linked immunosorbent assays (eBioScience, San Diego, CA, USA).

#### 2.5.4. Microvascular Endothelial Function

Skin microvascular flux was measured in arbitrary perfusion units (PU) using a DRT4 LDF device and MoorSoft for Windows/DRT4 v1.2 (Moor Instruments Ltd., Axminster, UK). All participants were placed in a supine position. The test was performed in a room with a controlled temperature (20–22 °C). Initially, a pneumatic cuff was placed on the distal part of the upper arm. Secondly, an LDF probe was placed on the ventral face of the forearm (at least 6 cm apart from the cuff). Additionally, to reduce physiological variability, the skin temperature around the probe was heated to a thermoneutral temperature (33 °C) using a Skin Heater Unit (Moor Instruments Ltd.). After a 5 min resting period, the basal microvascular flux was recorded for three minutes. Next, arterial occlusion was induced for three minutes (transient ischemia) by inflating the cuff at least 10 mmHg above the SBP. Finally, at the end of the occlusion period, the cuff was deflated, and PORH was then recorded over three minutes. Characterization of microvascular PORH was performed using a previously reported algorithm [28], which was developed in Matlab 9.5 (R2018b) (Mathworks Inc., Natick, MA, USA). The following parameters were calculated: basal flow (BF); peak flow (PF); and half-recovery flow (HRF), defined as (BF + PF)/2.

#### 2.5.5. Statistical Analysis

The sample size was calculated using MedCalc v13 considering the PF as the main outcome during post-occlusive reactive hyperemia [5]. Based on data from previous studies of our group [28,29], we expected to find an increase in PF around 25%, from a baseline level of 60 ± 15 PU. Thus, to detect a significant difference of 15 PU in PF between the two interventions (i.e., purple garlic oil and placebo), a total of 52 participants (26 per group) were required, considering a statistical power of 80% and a confidence level of 95%.

Continuous data were expressed as mean ± standard deviation (SD) or median [interquartile range (IQR)], according to variable distribution. Categorical variables were described in terms of frequency (%). Prior to hypothesis testing, the normality of the variables was assessed by the Shapiro–Wilk test and graphical representation. Between-groups differences in changes after five weeks were evaluated using Student’s *t*-test for independent samples or the U-Mann–Whitney test, as appropriate. Within-group differences were assessed using the paired-sample Student’s *t*-test or the Wilcoxon signed-rank test, according to normality. We considered *p* < 0.05 to be statistically significant. The analysis was performed using IBM-SPSS Statistics v24.0 and R v4.1.2.

## 3. Results

### 3.1. Subjects

**Figure 1:**
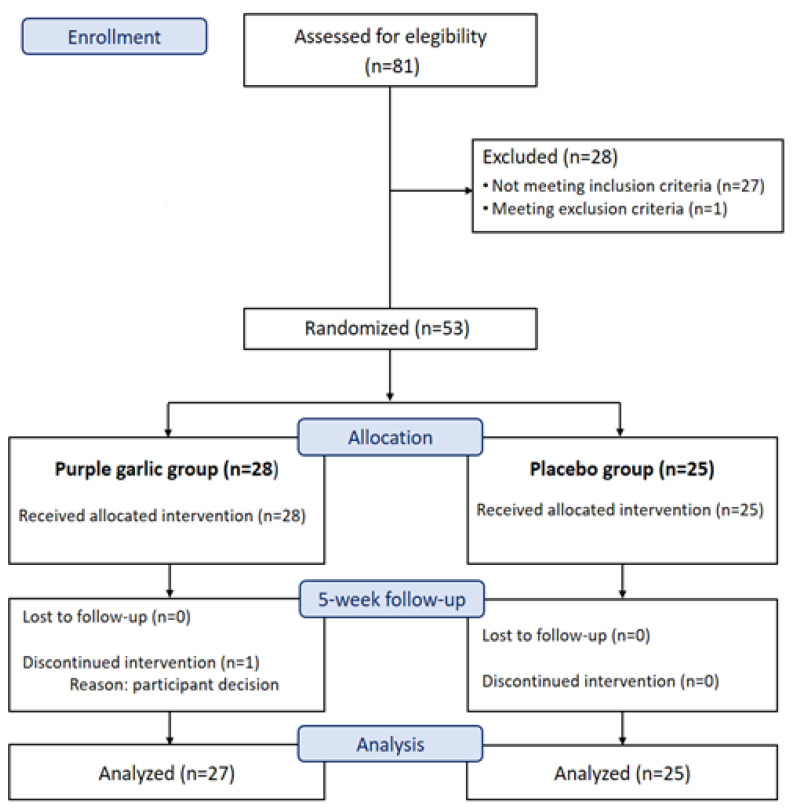
CONSORT flow chart of the study.

A total of 81 candidates were screened for the ENDOTALLIUM Study. Of these, 53 subjects met the eligibility criteria and were randomly assigned to the purple garlic oil arm or the placebo arm. One patient in the purple garlic group chose to drop out of the study during the intervention period. Finally, data from the n = 52 participants who completed the study protocol were included in the present analysis (Figure 1).

The mean age of the participants was 45.8 ± 13.6 years, ranging from 21 to 65 years old; most were female (59.6%). In our population, the prevalence of MetS was 23.1%, while 55.8% of the participants had only one MetS component. Elevated blood pressure was the most prevalent MetS component (51.9%), followed by abdominal obesity (42.3%). No significant differences in baseline characteristics were observed across treatment groups (Table 1).

### 3.2. Changes in Microvascular Function and Circulating Biomarkers

Regarding microvascular function, a significant increase in PF during PORH was observed in the purple garlic oil group after five weeks, compared to the control group (between-group mean difference [95% CI]: 15.4 [1.5 to 29.4] PU; *p* = 0.031). Likewise, HRF significantly increased in the intervention group when compared to placebo (between-group difference: 8.5 [0.9 to 16.2] PU; *p* = 0.029). In turn, after five weeks, BF was raised in subjects consuming purple garlic oil (14.4 (5.0) vs. 17.3 (6.8) PU; *p* = 0.017), but not in those in the placebo group, even though the between-group difference was not statistically significant (Table 2).

With regard to endothelial-related circulating biomarkers, we found that hs-CRP significantly decreased after five weeks in the purple garlic group compared to the placebo (between-group difference: −1.3 [−2.5 to −0.0] mg/L; *p* = 0.049). Meanwhile, VCAM-1 significantly decreased in both groups at week five, but the between-group difference was not statistically significant (26.3 [−41.9 to 94.5]). In turn, no significant within- and between-group differences were found in PAI-1 and urinary F2-isoprostanes/creatinine (Table 2).

### 3.3. Changes in MetS Components

**Figure 2:**
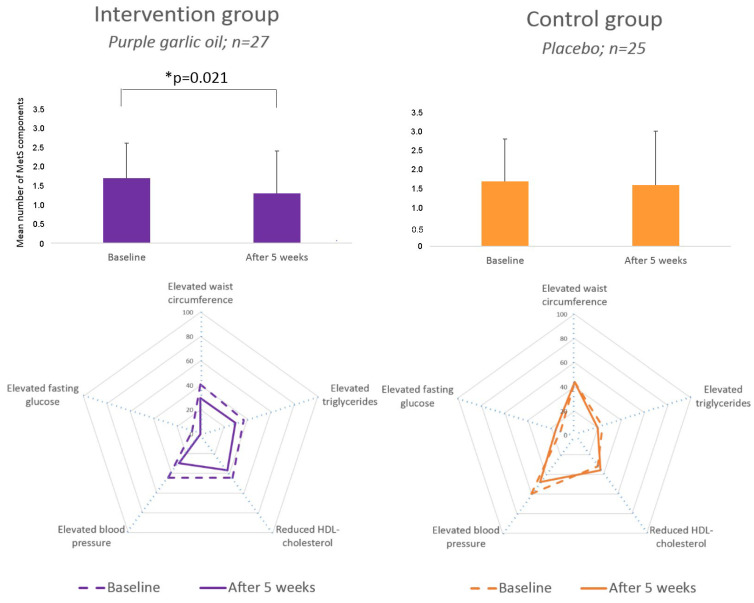
Changes in the overall metabolic syndrome (MetS) profile during the study (**left**: purple garlic oil; **right**: placebo). Each axis represents the prevalence (%) of a MetS component. The dashed line represents prevalences of MetS components at baseline and solid lines represent prevalences after five weeks. Within-group differences were evaluated using the paired-sample *t*-test.

After five weeks, a significant reduction in the mean number of MetS components was observed in the purple garlic group (1.7 ± 0.9 vs. 1.3 ± 1.1, *p* = 0.021), while no significant changes were observed in the control group (1.7 ± 1.1 vs. 1.6 ± 1.4, *p* = 0.538). The number of subjects with MetS in the intervention group decreased from 7/27 (25.9%) to 5/27 (18.5%), while in the control group changed from 5/25 (20.0%) to 6/25 (24.0%), albeit both differences were not statistically significant. Likewise, the prevalence of each MetS component tended to decrease in participants consuming purple garlic for five weeks, resulting in an improved overall MetS profile, as shown graphically (Figure 2). In turn, in the control group, the MetS profile remained similar during the intervention. Accordingly, the levels of several MetS parameters tended to improve in the purple garlic oil group (Table 3): fasting plasma glucose (−3.7 [−7.6 to 0.1] mg/dL; *p* = 0.054), triglycerides (−17.0 [−41.4 to 7.4]; *p* = 0.168), and HDL-cholesterol (2.3 [−0.7 to 5.3] mg/dL; *p* = 0.126). Furthermore, other parameters related to the lipid profile were similar at baseline, including total cholesterol (216.5 ± 42.7 vs. 209.6 ± 40.5 mg/dL) and LDL cholesterol (140.2 ± 38.5 vs. 134.1 ± 37.3 mg/dL), with no significant changes post-intervention.

### 3.4. Tolerability and Adverse Events

Regarding the tolerability of the investigational product, only 1 out of the 27 participants in the purple garlic oil arm discontinued the intake. The withdrawal was the subject’s decision and was related to the taste of the product. None of the participants in the placebo group discontinued the intake.

A total of five subjects presented adverse events throughout the study (two in the intervention group and three in the control group), but none of the adverse events were serious. Gastrointestinal symptoms, including nausea and diarrhea, were the most common adverse events: 5/7 (71.4%); two occurred in the purple garlic group and three in the control group. In addition, two subjects in the purple garlic oil arm reported experiencing a headache combined with gastrointestinal symptoms.

## 4. Discussion

To the best of our knowledge, the ENDOTALLIUM is the first study to assess the effects of purple garlic oil on the microvascular function, endothelial-related biomarkers, and the components of the MetS in untreated subjects with cardiometabolic alterations. This study has shown evidence of the beneficial effect of the regular consumption of encapsulated purple garlic oil on the skin microvascular function at early stages of the atherothrombotic disease.

In the present study, purple garlic oil was associated with an improvement in skin microvascular perfusion. Here, we show that subjects consuming encapsulated purple garlic oil for five weeks exhibited significantly improved skin blood flow at rest and, more importantly, during PORH, compared to placebo. Previous studies showed that microvasculature responds to cardiometabolic drugs such as statins, angiotensin-converting enzyme inhibitors, or insulin [30,31,32]. Moreover, current evidence supports favorable endothelial effects of healthy dietary patterns and plant-based foods [33]. In particular, the Mediterranean diet, whose components include garlic, has been linked to an improved microvascular response in populations with different cardiovascular risk factors [34,35,36,37]. Recently, the CORDIOPREV study [35], a randomized, double-blind, controlled trial, showed that long-term dietary intervention with either the Mediterranean diet or a low-fat diet significantly improved skin microvascular endothelial function in over 1000 coronary patients. Regarding garlic-derived products, Lindstedt et al. [38] reported a significant increase of 21.6% in the relative change of PORH in patients with atherosclerosis consuming aged garlic extract for 12 months. This result is in line with our findings, as we observed an increase of 18.8% in PORH in the purple garlic oil group, even after a shorter intervention period.

Garlic oil may improve microvascular function due to the antioxidant and anti-inflammatory activity of its sulfur compounds, such as DAS, DADS, and DATS [18]. Underlying mechanisms that may account for these beneficial effects could be related to its ability to scavenge reactive oxygen species and reduce inflammation by regulating interleukin-10 [39,40]. Notably, our study has shown a significant decrease in CRP levels, a systemic inflammatory mediator and an acute phase reactant that has been positively associated with cardiovascular disease risk and endothelial dysfunction [41]. In this regard, a meta-analysis on the effects of garlic supplementation on serum inflammatory markers proved that consuming garlic significantly reduces the CRP [39]. Regarding the antioxidant activity, some previous studies had described an improvement in total antioxidant capacity and lipid peroxidation products in a variety of cohorts after regular consumption of garlic preparations, such as aged garlic extract [42] or garlic powder [43], but these findings have not been replicated consistently [44]. In our study, urinary F2-isoprostanes/creatinine levels, a surrogate biomarker of oxidative stress, did not change after five weeks of intervention. These results, combined with those of VCAM-1 and PAI-1, could suggest that a longer period of administration may be necessary to have an effect on circulating biomarkers of oxidative stress and endothelial dysfunction in untreated subjects with mild cardiometabolic alterations. Nevertheless, our functional and biochemical findings jointly provide evidence for a protective role of purple garlic oil on endothelium.

Several studies have demonstrated a beneficial effect of garlic and its compounds against various disorders such as hypertension, hyperlipidemia, and diabetes, all of which are key components of MetS [45,46,47,48,49]. The ENDOTALLIUM Study has shown that consuming purple garlic oil for five weeks was associated with a significant decrease in the mean number of MetS components. In this regard, Girkantaite et al. reported that the number of components of the MetS inversely correlates with skin microvascular endothelial function [50]. Thus, the enhanced microvascular function reported in the current study is consistent with the significant decrease in the number of MetS components. However, in contrast to previous studies [51,52], we failed to show statistically significant differences in MetS-related parameters.

Regarding safety and tolerability, we observed few adverse events, mainly mild gastrointestinal symptoms, with similar incidences in garlic oil and placebo groups. Our findings are consistent with previous evidence [53,54] which reported that adverse effects associated with garlic oil consumption are uncommon, with most of them being non-specific and related to gastrointestinal discomfort or nausea [25]. Noteworthy, in our study the purple garlic oil underwent two encapsulation processes, which may have contributed to mitigate the odor and taste of garlic oil, improving tolerability and adherence to its consumption. Likewise, the encapsulation could play a role in enhancing the stability and bioaccessibility of the organosulfur compounds in garlic oil [55]. In sum, our results support the favorable risk–benefit profile of garlic oil supplementation for individuals with cardiometabolic alterations.

Our findings should be interpreted in the light of some limitations. First, we chose a population at an early stage of cardiovascular disease, who fulfilled at least one of the MetS components. It should be noted that most of the participants did not have a clinical diagnosis of MetS (characterized by three or more MetS components) [23]. This could have limited the ability to achieve significant changes in some of the MetS-related parameters, since their baseline levels were only slightly impaired. Likewise, we excluded patients undergoing pharmacological treatment for the main cardiovascular risk factors to avoid the confounding effect of these drugs on the endothelial-related biomarkers and the microvascular function. Therefore, caution should be exercised when generalizing our findings to patients at more advanced stages of the cardiovascular continuum. Second, the intervention period was limited to five weeks to balance the potential benefits and risks, given that this was the first clinical study involving this garlic-derived product. Nevertheless, longer exposure is needed to demonstrate long-term effects. Third, although we controlled dietary intake of antioxidants, our study took place in a Mediterranean country where, on average, the diet is rich in fruits and vegetables, including garlic. Fourth, we used PORH coupled with LDF to assess skin microvascular function. Reactive hyperemia represents a complex microvascular response to an acute period of ischemia, in which the endothelium has a potential role but acts independently of NO. Unlike the macrovascular endothelial function, which can be assessed by more standardized techniques as the flow-mediated dilatation of the brachial artery, microvascular response measured by LDF has often been considered poorly reproducible [5]. Hence, we standardized the site of measurement and controlled skin temperature to minimize the variability of the technique. Finally, this study was designed as a single-blinded study, in which only the investigator was aware of the group assignment, which could increase the likelihood of bias compared to a double-blinded design.

## 5. Conclusions

Regular consumption of encapsulated purple garlic oil significantly improved microvascular function in a population with cardiometabolic alterations. Additional benefits were also observed in subclinical inflammatory status and the components of MetS. Thus, purple garlic oil may play a potential role in the management of cardiometabolic disorders at early stages, when pharmacological treatment is not yet indicated. Nevertheless, further research is needed to establish the long-term benefits of purple garlic oil in microvasculature in different patient populations.

## Figures and Tables

**Table 1:** Baseline characteristics of the participants.

	Intervention Group Purple Garlic Oil (*n* = 27)	Control Group Placebo (*n* = 25)
Demographic characteristics		
Age, mean ± SD, years	46.0 ± 12.9	45.7 ± 14.7
Gender, n (%)		
Female	17 (63.0%)	14 (56.0%)
Male	10 (37.0%)	11 (44.0%)
Clinical characteristics related to Metabolic Syndrome (NCEP/ATP III criteria) [23]		
Metabolic Syndrome, n (%)	7 (25.9%)	5 (20.0%)
No. of Metabolic Syndrome components, n (%)		
1	14 (51.9%)	15 (60.0%)
2	6 (22.2%)	5 (20.0%)
3	7 (25.9%)	3 (12.0%)
4–5	0 (0.0%)	2 (8.0%)
Metabolic Syndrome components, n (%)		
Elevated waist circumference	11 (40.7%)	11 (44.0%)
Elevated triglycerides	10 (37.0%)	6 (24.0%)
Reduced HDL-cholesterol	12 (44.4%)	8 (32.0%)
Elevated blood pressure	12 (44.4%)	15 (60.0%)
Elevated fasting glucose	2 (7.4%)	3 (12.0%)
Lifestyle habits		
Current smoker, n (%)	6 (22.2%)	5 (20.0%)
Physical activity status ^a^, n (%)		
Low physical activity	3 (11.1%)	5 (20.0%)
Moderate physical activity	15 (55.6%)	11 (44.0%)
High physical activity	9 (33.3%)	9 (36.0%)
Dietary habits, mean ± SD		
Fruits and vegetables, servings/day	3.0 ± 0.9	2.8 ± 1.1
Nuts and dried fruits, servings/week	3.3 ± 2.9	4.4 ± 4.5
Garlic, cloves/week	2.0 ± 2.2	3.3 ± 2.0

Characteristics collected at inclusion. Data expressed as n (%) or mean ± standard deviation. ^a^ Physical activity categorized according to the physical activity rating scale (PA-R): low (PA-R from 0 to 1), moderate (PA-R from 2 to 3), and high (PA-R ≥ 4) [27].

**Table 2:** Skin microvascular function and endothelial-related circulating biomarkers at baseline and changes after 5 weeks.

	Intervention Group Purple Garlic Oil (*n* = 27)	Control Group Placebo (*n* = 25)	Between-Group Difference [95% CI]	*p*-Value Between-Group Differences
Skin microvascular function				
Basal flow (BF), mean ± SD, PU				
Baseline Change after 5 weeks	14.4 ± 5.0 +2.9 ± 5.4 *	15.6 ± 6.0 +1.2 ± 7.5	1.7 [−2.2 to 5.6]	0.393
Occlusion flow, mean ± SD, PU				
Baseline Change after 5 weeks	4.9 ± 1.0 −0.3 ± 0.9	5.3 ± 1.5 −0.2 ± 1.9	−0.2 [−1.1 to 0.8]	0.722
Peak flow (PF), mean ± SD, PU				
Baseline Change after 5 weeks	61.1 ± 19.6 +11.5 ± 23.7 *	71.7 ± 24.5 −3.9 ± 22.6	15.4 [1.5 to 29.4]	0.031
Half-recovery flow (HRF), mean ± SD, PU				
Baseline Change after 5 weeks	37.9 ± 11.0 +7.2 ± 12.8 *	43.7 ± 13.5 −1.3 ± 12.6	8.5 [0.9 to 16.2]	0.029
Endothelial-related circulating biomarkers				
hs-CRP, mg/L				
Baseline, median [IQR] Change after 5 weeks, mean ± SD	2.9 [2.8] −0.5 ± 2.1	2.5 [1.9] +0.8 ± 2.1	−1.3 [−2.5 to −0.0]	0.049
VCAM-1, mean ± SD, ng/mL				
Baseline Change after 5 weeks	906.4 ± 182.2 −41.2 ± 129.4 *	1028.5 ± 288.0 −67.5 ± 114.3 *	26.3 [−41.9 to 94.5]	0.443
PAI-1, ng/mL				
Baseline, median [IQR] Change after 5 weeks, mean ± SD	38.5 [41.2] +1.6 ± 19.8	30.9 [34.4] +5.8 ± 16.5	−4.2 [−14.4 to 6.0]	0.414
Urinary F2-isoprostanes/creatinine, mmol/mg				
Baseline, median [IQR] Change after 5 weeks, mean ± SD	132.3 [90.3] −6.5 ± 92.0	97.5 [75.1] −10.9 ± 75.0	4.5 [−51.8 to 42.9]	0.851

Data expressed as mean (standard deviation) or median [interquartile range]; 95% CI: 95% confidence interval (CI) for between-group differences; SD: standard deviation; IQR: interquartile range; hs-CRP: high-sensitivity C-reactive protein; VCAM-1: vascular cell adhesion molecule-1; PAI-1: plasminogen activator inhibitor-1; PU: arbitrary perfusion units. * *p* < 0.05 for within-group differences using the paired-sample Student’s *t*-test, as all mean differences met the normality assumption.

**Table 3:** Metabolic syndrome parameters at baseline and changes after 5 weeks.

MetS Parameters	Intervention Group Purple Garlic Oil (*n* = 27)	Control Group Placebo (*n* = 25)	Between-Group Difference [95% CI]	*p*-Value
Waist circumference, mean ± SD, cm				
Baseline Change after 5 weeks	88.9 ± 9.5 −0.4 ± 3.3	88.6 ± 11.0 −0.4 ± 4.6	0.0 [−2.2 to 2.3]	0.977
Triglycerides, mean ± SD, mg/dL				
Baseline Change after 5 weeks	132.3 ± 54.1 −14.1 ± 40.8	111.6 ± 57.3 +2.9 ± 46.7	−17.0 [−41.4 to 7.4]	0.168
HDL-cholesterol, mean ± SD, mg/dL				
Baseline Change after 5 weeks	49.6 ± 11.2 +1.0 ± 5.6	52.8 ± 13.7 −1.3 ± 5.1	2.3 [−0.7 to 5.3]	0.126
SBP, mean ± SD, mmHg				
Baseline Change after 5 weeks	124.2 ± 14.7 −2.2 ± 7.5	129.1 ± 16.0 −3.0 ± 11.5	0.8 [−4.6 to 6.1]	0.773
DBP, mean ± SD, mmHg				
Baseline Change after 5 weeks	78.1 ± 8.7 −1.0 ± 4.4	79.7 ± 5.9 −1.0 ± 5.5	0.0 [−2.8 to 2.8]	0.998
Fasting glucose, mean ± SD, mg/dL				
Baseline Change after 5 weeks	81.9 ± 8.1 −1.1 ± 7.2	80.6 ± 12.4 +2.6 ± 6.4	−3.7 [−7.6 to 0.1]	0.054

Data expressed as mean (standard deviation) or median [interquartile range]. 95% CI: 95% confidence interval (CI) for between-group differences; MetS: metabolic syndrome; DBP: diastolic blood pressure; SBP: systolic blood pressure; SD: standard deviation.

## Data Availability

The data presented in this study are available on request from the corresponding author. The data are not publicly available due to participant confidentiality.
